# The Endocrine Disruptor Mono-(2-Ethylhexyl) Phthalate Affects the Differentiation of Human Liposarcoma Cells (SW 872)

**DOI:** 10.1371/journal.pone.0028750

**Published:** 2011-12-21

**Authors:** Enrico Campioli, Amani Batarseh, Jiehan Li, Vassilios Papadopoulos

**Affiliations:** 1 Research Institute of the McGill University Health Center and the Departments of Medicine, Biochemistry, and Pharmacology and Therapeutics, McGill University, Montréal, Québec, Canada; 2 Department of Biomedical Sciences, University of Modena and Reggio Emilia, Modena, Italy; Laurentian University, Canada

## Abstract

Esters of phthalic acid (phthalates) are largely used in industrial plastics, medical devices, and pharmaceutical formulations. They are easily released from plastics into the environment and can be found in measurable levels in human fluids. Phthalates are agonists for peroxisome proliferator-activated receptors (PPARs), through which they regulate translocator protein (TSPO; 18 kDa) transcription in a tissue-specific manner. TSPO is a drug- and cholesterol-binding protein involved in mitochondrial respiration, steroid formation, and cell proliferation. TSPO has been shown to increase during differentiation and decrease during maturation in mouse adipocytes. The purpose of this study was to establish the effect of mono-(2-ethylhexyl) phthalate (MEHP) on the differentiation of human SW 872 preadipocyte cells, and examine the role of TSPO in the process. After 4 days of treatment with 10 µM MEHP, we observed changes in the transcription of acetyl-CoA carboxylase alpha, adenosine triphosphate citrate lyase, glucose transporters 1 and 4, and the S100 calcium binding protein B, all of which are markers of preadipocyte differentiation. These observed gene expression changes coincided with a decrease in cellular proliferation without affecting cellular triglyceride content. Taken together, these data suggest that MEHP exerts a differentiating effect on human preadipocytes. Interestingly, MEHP was able to temporarily increase TSPO mRNA levels through the PPAR-α and β/δ pathways. These results suggest that TSPO can be considered an important player in the differentiation process itself, or alternatively a factor whose presence is essential for adipocyte development.

## Introduction

Phthalates are esters of phthalic acid largely used in industrial plastics to increase the flexibility of polymers, household items, and medical devices, and in pharmaceutical formulations such as stabilizers, lubricants, and emulsifying agents [1]. Due to a lack of covalent bonds between plastics and phthalates, phthalates are easily released from plastics into the environment [2], [3]. According to the United States Environmental Protection Agency, the United States Agency for Toxic Substances and Disease Registry, and the Medical Devices Bureau of Health (Canada), the largest exposure of the general population to di-(2-ethylhexyl) phthalate (DEHP) occurs via food, followed by indoor air contamination [4], [5]. The range of exposure in the general population (excluding medical and occupational sources) is estimated at 3–30 µg/kg body weight/day, and infants and toddlers are the most exposed (18.9 and 25.8 µg/kg body weight/day, respectively) [4]–[7]. Phthalates are rapidly metabolized and excreted in urine and feces [8], but are found in measurable levels in blood, semen, and breast milk [9]–[11].

Mono-(2-ethylhexyl) phthalate (MEHP), the most toxic metabolite of DEHP, is a well-known ligand for the peroxisome proliferator-activated receptor (PPAR) family, as are all other phthalates. PPARs are nuclear receptors that act as transcription factors of genes involved in lipid and glucose metabolism [12]–[14]. At present 3 isoforms have been identified, named PPAR-α, β/δ, and γ. The expression of PPAR-α was traditionally associated with the liver [15], where it is responsible for lipid catabolism [13], [14]. However, it was recently demonstrated that PPAR-α can upregulate β-oxidation and decrease glucose utilization in human white adipocytes [16]. PPAR-β/δ is ubiquitous and has been observed to play a role in the regulation of energy homeostasis in skeletal muscle [17], and in keratinocyte proliferation and differentiation during wound healing [18]. PPAR-γ is the major isoform found in adipose tissue, but it is also expressed at high levels in the spleen and digestive tract [15]. Its main functions in the adipose tissue are the storage of lipids [14], [19] and the regulation of adipocyte differentiation [14], [20], [21]. More generalized effects of PPAR-γ have also been observed; its agonists (thiazolidinediones) have been shown to lower blood pressure [22] and exert an antitumor effect in many cell lines and organs [23].

In a previous study, we demonstrated that phthalates decrease the levels of testicular translocator protein (TSPO; 18 kDa) mRNA and of circulating testosterone in mice through the action of PPAR-α, and increase TSPO levels in the liver [24]. TSPO, previously known as peripheral-type benzodiazepine receptor (PBR) [25], is part of a multimeric protein complex that is located in the outer mitochondrial membrane (OMM). TSPO is quite ubiquitous, but it is expressed in high levels in steroidogenic tissues, where it exerts its main function of cholesterol transport into mitochondria, and supports steroid biosynthesis. Besides cholesterol transport into mitochondria, TSPO is involved in many cellular functions, such as mitochondrial respiration, mitochondrial permeability transition pore opening, apoptosis, and cell proliferation [25], [26]. We have shown that protein kinase C ε (PKCε) affects TSPO expression through the mitogen-activated protein kinase (MAPK) pathway [27]. In cell lines expressing low endogenous levels of TSPO, stimulation by phorbol 12-myristate 13-acetate (PMA) has been demonstrated to cause an increase in TSPO levels [28]. The expression of TSPO in 3T3-L1 preadipocyte cell lines, and the upregulation of TSPO mRNA during adipogenesis, has been demonstrated [29]; however, further information about the role of TSPO in adipose tissue is lacking. TSPO was previously detected by a radioligand binding assay in the interscapular brown adipose tissue [30] and in the epididymal adipose tissue where its expression is increased in response to acute stress [31].

The aim of the present study was to evaluate the effect of MEHP on the differentiation of human preadipocytes from the human liposarcoma-derived SW 872 cell line, and investigate the relationship between the PPAR family and TSPO.

## Methods

### Cell Culture

Human liposarcoma SW 872 cells were purchased from American Type Culture Collection (ATCC, Manassas, VA, USA), cultured in 75-cm^2^ flasks (Dow Corning Corp., Corning, NY, USA) in a humidified atmosphere with 3.5% CO_2_ at 37°C, in Dulbecco's Modified Eagle Medium: Nutrient Mixture F-12 (Invitrogen Canada Inc., Burlington, ON, Canada) supplemented with 10% fetal bovine serum (Invitrogen Canada Inc.). SW 872 cells differentiate over time in culture without the use of any differentiation media [32]. Indeed, SW 872 are preadipocytes which stop proliferating when they reach 100% confluence. It is at this point when they begin differentiating into adipocytes.

### Quantification of mRNA levels by quantitative real time-polymerase chain reaction (qRT-PCR)

SW 872 cells were plated onto 6-well plates (Dow Corning Corp.) at the initial concentration of 7.5×10^4^ cells per well. After 1 day (60% confluence, D0), the cells were treated with 1, 10, and 50 µM of MEHP and 10, 50, and 100 nM of PMA (all purchased from Sigma-Aldrich Canada Ltd., Oakville, ON, Canada) for a period of 4, 8, or 12 days.

Total RNA was extracted from cultured cells using RNeasy Mini Kit (Qiagen Inc., Mississauga, ON, Canada) according to manufacturer's instructions. The RNA concentration was determined by measuring absorbance at 260 nm using the NanoDrop ND-1000 (Thermo Scientific, Mississauga, ON, Canada). The purity of RNA samples was determined by the A260/A280 (>2.0) and the A260/A230 (2.0–2.2) values.

Samples were normalized to total RNA content and then reverse transcribed using Transcriptor First Strand cDNA Synthesis Kit (Roche Applied Science, Laval, QC, Canada) according to manufacturer's instructions. The resulting cDNA samples were diluted with nuclease-free water and subjected to qRT-PCR using the SYBR green dye technique on a Light Cycler system 480 (Roche Applied Science). Briefly, samples were pre-incubated at 95°C for 5 min followed by 45 cycles of denaturation at 95°C for 10 s, annealing at 61°C for 10 s and elongation at 72°C for 10 s.

Following PCR amplification, the identity of the amplification product was verified by electrophoresis on a 2% agarose gel and by melting curve analysis. The results reported for each RNA product have been normalized to ribosomal protein S18 (RPS18) mRNA to correct for differences in the amounts of the template cDNA. Oligonucleotide sequences of sense and antisense primers are shown in Table 1.

**Table 1:** Primers used for qRT-PCR analysis.

mRNA	Forward primer 5′-3′	Reverse primer 5′-3′
**ACACA**	CGGGAGAAGAGGGAATTGGACCCG	TAGGCCAATGAGGATTCTCCAG
**ACLY**	CGGACCGAAGTCACAATCTTTGTCCG	GGTCTTCCCGACTTCTCCCATC
**C/EBP-α**	CGGTCATTGTCACTGGTCAGC	CGTTGGGTGGACAAGAACAGCAACG
**GLUT1**	CGGAACTTCGATGAGATCGCTTCCG	AACAGCTCCTCGGGTGTCTTG
**GLUT4**	GGGCCTGCCAGAAAGAGTCTG	CGATGCCAGCACTCCAGAAACATCG
**PKC_ε_**	CTGCCTTTGCCCAACACCTT	CGGACGGTGAGAATGGCGAAGTCCG
**PPAR-α**	TTCCTGCAAGAAATGGGAAACA	CGGCTTCCAGAACTATCCTCGCCG
**PPAR-β/δ**	GGCAGCCTCAACATGGAGTG	CGGGAACACCGTAGTGGAAGCCCG
**PPAR-γ**	ATGGAGTTCATGCTTGTGAAGGA	CGGAGGCTTCAATCTGATTGTTCTCCG
**RPS18**	CGGGCATCCTCAGTGAGTTCTCCCG	TCATGTGGTGTTGAGGAAAGCA
**S100B**	CACGAGCACTCATGTTCAAAGAACTCGTG	ATGGAGACGGCGAATGTGACT
**TSPO**	GGTGGATCTCCTGCTGGTCA	CGACCAATACGCAGTAGTTGAGTGTG

ACACA, Acetyl-CoA carboxylase alpha; ACLY, ATP citrate lyase; C/EBP-α, CCAAT/enhancer binding protein alpha; GLUT1, Glucose transporter 1; GLUT4, Glucose transporter 4; PKCε, protein kinase C ε; PPAR-α, peroxisome proliferator-activated receptor alpha; PPAR-β/δ, peroxisome proliferator-activated receptor beta/delta; PPAR-γ, peroxisome proliferator-activated receptor gamma; RPS18, ribosomal protein S18; S100B, S100 calcium binding protein B; TSPO, translocator protein 18-kDa.

### Triglyceride quantification assay

Triglyceride concentration was measured with a colorimetric assay. Briefly, cells were seeded in 75-cm^2^ flasks (Dow Corning Corp.) and were maintained for 24 h to allow adherence. The culture medium was supplemented with 10 µM of MEHP (Sigma-Aldrich Canada Ltd.) for 4 or 6 days; then samples containing 1×10^7^ cells each were homogenized in 1 ml 5% Triton-X100, and slowly heated to 90°C in a water bath for 5 min. After a second heating, samples were centrifuged and the supernatant was diluted 10-fold with distilled water. The Triglyceride Quantification assay (Abcam, Cambridge, MA, USA) was performed according to the manufacturer's instructions and the reaction was quantified by spectrophotometer at 595 nm using a VICTOR™ X5 Multilabel Plate Reader (PerkinElmer Inc., Waltham, MA, USA). The triglyceride concentration (µM/10^7^ cells) was calculated according to the formula C = T_s_/S_v_; where C is the concentration, T_s_ is the triglyceride amount from the standard triglyceride curve, and S_v_ is the sample volume in the well.

### Bromodeoxyuridine (BrdU) proliferation assay

Cell proliferation was evaluated using the BrdU assay (Roche Applied Science), a colorimetric immunoassay based on the incorporation of BrdU during DNA synthesis in proliferating cells. Briefly, cells were seeded in 96-well plates (Dow Corning Corp.) at the initial concentration of 2000 cells/well and were maintained for 24 h to allow adherence. The culture medium was supplemented with 10 µM of MEHP (Sigma-Aldrich Canada Ltd.) for 3 days. Following this, BrdU solution was added to the well for 8 h, then the medium was aspirated and cells were fixed with FixDenat (provided with the assay) for 30 min at room temperature. FixDenat was then removed and the samples were incubated for 90 min with anti-BrdU solution at room temperature. After washing the wells, the amount of immune complex in each well was quantified by spectrophotometric measurement at 490 nm and 690 nm with VICTOR™ X5 Multilabel Plate Reader (PerkinElmer Inc.). Results were obtained by subtracting absorption at 690 nm from the absorption at 490 nm for each sample. The subtracted absorbances were then normalized to control values and expressed as percent of BrdU incorporation.

### Transfection of small interfering RNA (siRNA)

Cells were plated onto 6-well plates (Dow Corning Corp.) at an initial concentration of 7.5×10^4^ cells per well, and immediately transfected with siRNA using Lipofectamine™ 2000 (Invitrogen Canada Inc.) according to manufacturer's instructions. PKCε (80 nM), PPAR-β/δ (40 nM), PPAR-γ (40 nM), and TSPO (40 nM) siRNAs were all part of the ON-TARGETplus SMARTpool range of siRNA products produced by Dharmacon Products, Thermo Fisher Scientific (Lafayette, CO, USA). PPAR-α siRNA (Silencer® validated siRNA, 40 nM) was purchased from Applied Biosystems Canada (Streetsville, ON, Canada), and a scrambled siRNA (ON-TARGETplus Non-Targeting siRNA, 40 nM) from Dharmacon was used as a transfection control. After 1 day (60% confluence, D0), the medium was changed and the cells were incubated with 10 µM MEHP for 4 days. Gene expression was evaluated as explained above in the section 2.2. Target gene knockdown was verified by qRT-PCR.

To validate the findings observed by gene silencing we treated the SW 872 cells with increasing concentrations (of 5, 10, 15 and 50 µM ) of the PKCε translocation inhibitor peptide H-Glu-Ala-Val-Ser-Leu-Lys-Pro-Thr-OH (EMD4Biosciences, EMD Inc, Mississauga, ON, Canada) for 4 days. At the end of the experiments cells were collected and TSPO mRNA levels were determined by qRT-PCR.

### Immunoblot analysis

Cells were incubated in a 10 cm^2^ dish for 4 days with 10 µM MEHP, then collected and lysed in 1× cold RIPA buffer (Cell Signaling, New England Biolabs Ltd., Pickering, ON, Canada). Protein extracts were obtained by centrifugation of the homogenate at 4°C, and the protein concentration was measured using the Bradford assay (Bio-Rad, Hercules, CA, USA). Proteins (30 µg) were electrophoretically separated on a 4–20% Tris-glycine SDS gradient gel (Invitrogen Canada Inc.), then transferred onto polyvinylidene fluoride membranes (Invitrogen Canada Inc.) and blocked for 1 h at room temperature in blocking buffer (20 nM Trizma Base, 100 mM NaCl, 1% Tween-20, 10% skim milk; Sigma-Aldrich Canada Ltd.). Membranes were then incubated overnight at 4°C with a rabbit immunoglobulin G (IgG) anti-TSPO polyclonal antibody (1∶300 dilution) [33]. To verify the uniformity of protein loading, each membrane was also incubated with anti-β-actin antibody (1∶2000 dilution; Cell Signaling). Finally, membranes were washed and incubated for 1 h at room temperature with a goat anti-rabbit IgG horseradish peroxidase (1∶2000 dilution; Cell Signaling). The complex was visualized using the Amersham chemiluminescence kit (GE Healthcare, Baie d'Urfe, QC, Canada), and a FUJI image reader LAS4000 (FUJIFILM Holdings America Corporation, Valhalla, NY, USA) for capturing images. The intensity of each band was measured using Multigauge V3.0 software (FUJIFILM Holdings America Corporation) and normalized to that of the corresponding β-actin band.

### [^3^H]PK11195 radioligand binding assay

In radioligand binding assays, increasing concentrations of [^3^H]PK11195 (100 µl, 0.39–12.5 nmol/l; specific activity 2.22–3.33 TBq/mmol; PerkinElmer Inc.) were incubated with membranes containing lysates from control and MEHP treated SW 872 cells (100 µl, 2–10 µg of protein/ml). Briefly, cell pellets were homogenized in phosphate buffered saline (Invitrogen Canada Inc.) with a glass Teflon homogenizer (Wheaton Science Products, Millville, NJ, USA). Protein concentration was determined by the Bradford method using bovine serum albumin as a standard. The final volume (300 µl) was obtained by adding 100 µl of water, and nonspecific binding was measured in the presence of unlabeled PK11195 (100 µl, 6.6 µmol/l; Sigma-Aldrich Canada Ltd.). The mixture was incubated at 0–4°C for 90 min. The reaction was terminated by filtering on glass filters (GF/B; Brandel Inc., Gaithersburg, MD, USA) presoaked in 0.5% polyethyleneimine (Sigma-Aldrich Canada Ltd.) using the Brandel binding apparatus (Brandel Inc.). The filters were transferred to vials with 3 ml of Ecolite(+)™ liquid scintillation cocktail (MP Biomedicals, Solon, OH, USA) and radioactivity was measured in an LS 5801 RackBeta liquid scintillation counter (Beckman Coulter Inc., Brea, CA, USA). Scatchard analysis of the saturation isotherms of all samples was performed using GraphPad (GraphPad Software, La Jolla, CA) to obtain the maximal number of receptors (B_max_), expressed as fmol/mg proteins, and dissociation constants (Kd).

### Statistical analysis

Data were expressed as mean, S.E.M., and n, and were analyzed using the Student's *t*-test, or one-way ANOVA followed by Bonferroni's post hoc test, using the GraphPad Prism program (GraphPad Software Inc., La Jolla, CA, USA). p<0.05 (*, ^#^), p<0.01 (**), and p<0.001 (***) were used as indicators of the level of significance.

## Results

### Basal mRNA levels of the differentiation markers PPARs, TSPO, and PKCε during SW 872 cell differentiation

Since there is little information on SW 872 cell differentiation markers in literature, we investigated the gene expression of the well-known differentiation markers CCAAT/enhancer binding protein alpha (C/EBP-α), glucose transporter 4 (GLUT4), S100 calcium binding protein B (S100B) [32], [34], PPAR-α, PPAR-β/δ, PPAR-γ, TSPO, and PKCε, at specific times after differentiation.

**Figure 1:**
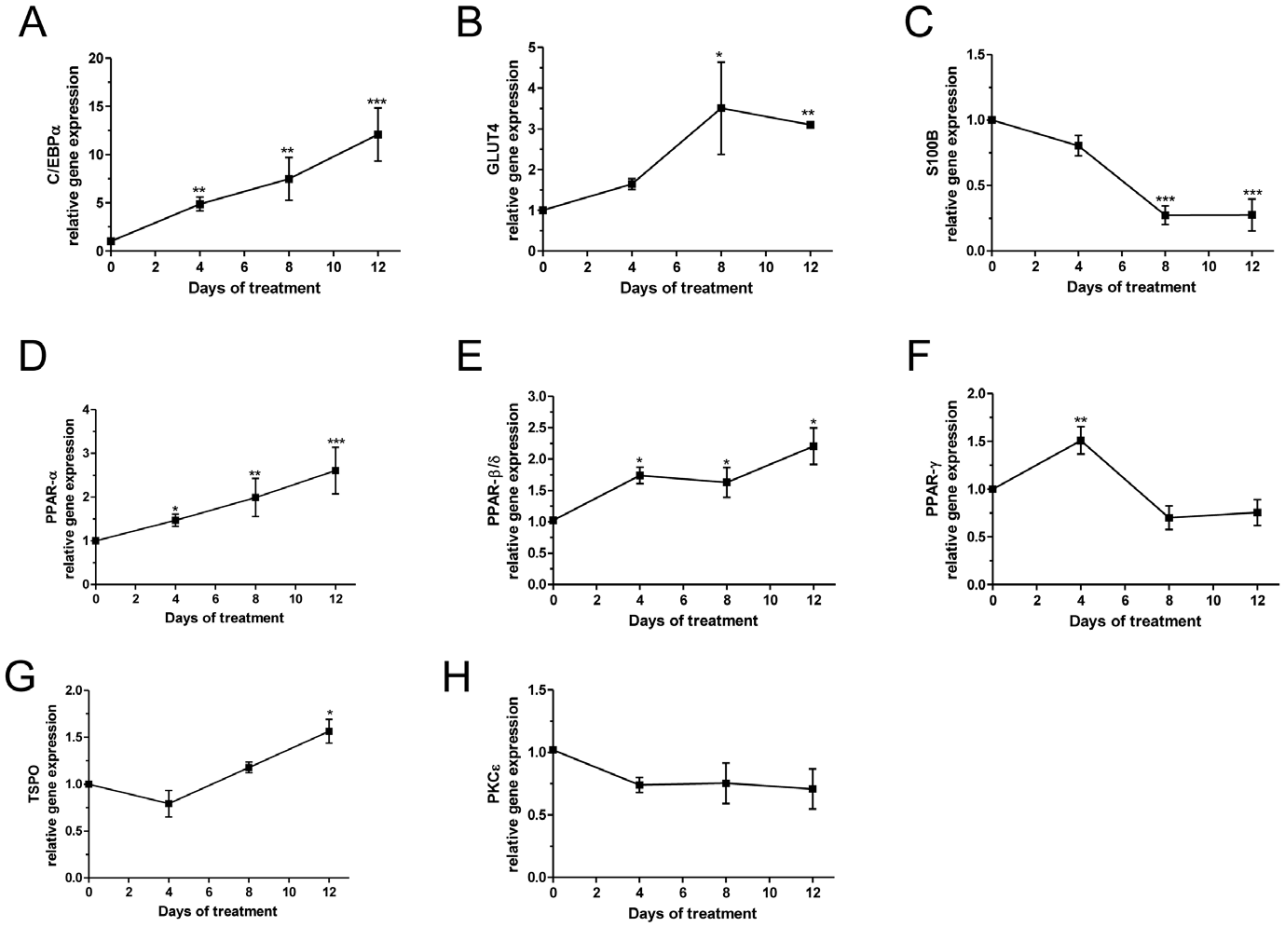
Basal levels of (A) C/EBP-α, (B) GLUT4, (C) S100B, (D) PPAR-α, (E) PPAR-β/δ, (F) PPAR-γ, (G) TSPO, (H) PKCε mRNAs normalized to RPS18. Cells were seeded and collected after the indicated time points; day 0 represents one day after the plating. Results are expressed in terms of mean and S.E.M., calculated from 3 independent experiments and presented as fold increase or decrease from the value measured on day 0. Significance (compared to day 0 values) was calculated using one-way ANOVA followed by Bonferroni's post hoc test; *p<0.05, **p<0.01, ***p<0.001.

At 4, 8, and 12 days after initiation of differentiation in cultured cells, C/EBP-α mRNA levels increased approximately 5-fold (p<0.01), 7.5-fold (p<0.01), and 12-fold (p<0.001), respectively (Fig. 1A) as previously described [32]. GLUT4 mRNA levels increased by approximately 3.5-fold (p<0.05) and 3-fold (p<0.01), 8 and 12 days after initiation of differentiation, respectively (Fig. 1B), while in those same time periods, S100B mRNA levels decreased by 73% (p<0.001) and 72% (p<0.001), respectively (Fig. 1C). At 4, 8, and 12 days after initiation of differentiation, PPAR-α mRNA levels showed an increase of approximately 1.5-fold (p<0.05), 2-fold (p<0.01), and 2.6-fold (p<0.001), respectively (Fig. 1D), while PPAR-β/δ mRNA increased by approximately 1.7-fold (p<0.05), 1.6-fold (p<0.05), and 2.2-fold (p<0.05), respectively (Fig. 1E). At 4 days after initiation of differentiation, PPAR-γ mRNA levels increased by approximately 1.5-fold (p<0.01; Fig. 1F), and subsequently, decreased progressively. This observation is in agreement with Carmel et al. [32], and suggests an inverse pattern compared to murine 3T3-L1 preadipocytes [20] where PPAR-γ mRNA was shown to progressively increase.

Because TSPO mRNA was found to be present and to increase during differentiation in murine preadipocytic 3T3-L1 cells [29], we also investigated *TSPO* gene expression during the differentiation of SW 872 cells. We observed an approximate 1.6-fold increase in *TSPO* mRNA levels (p<0.05) after 12 days of differentiation (Fig. 1G), consistent with the increased TSPO expression observed by Wade et al. during the differentiation of 3T3-L1 mouse preadipocytes [29]. No significant changes were observed in PKCε transcription during the differentiation of human SW 872 cells into adipocytes (Fig. 1H).

### MEHP increases TSPO mRNA levels

**Figure 2:**
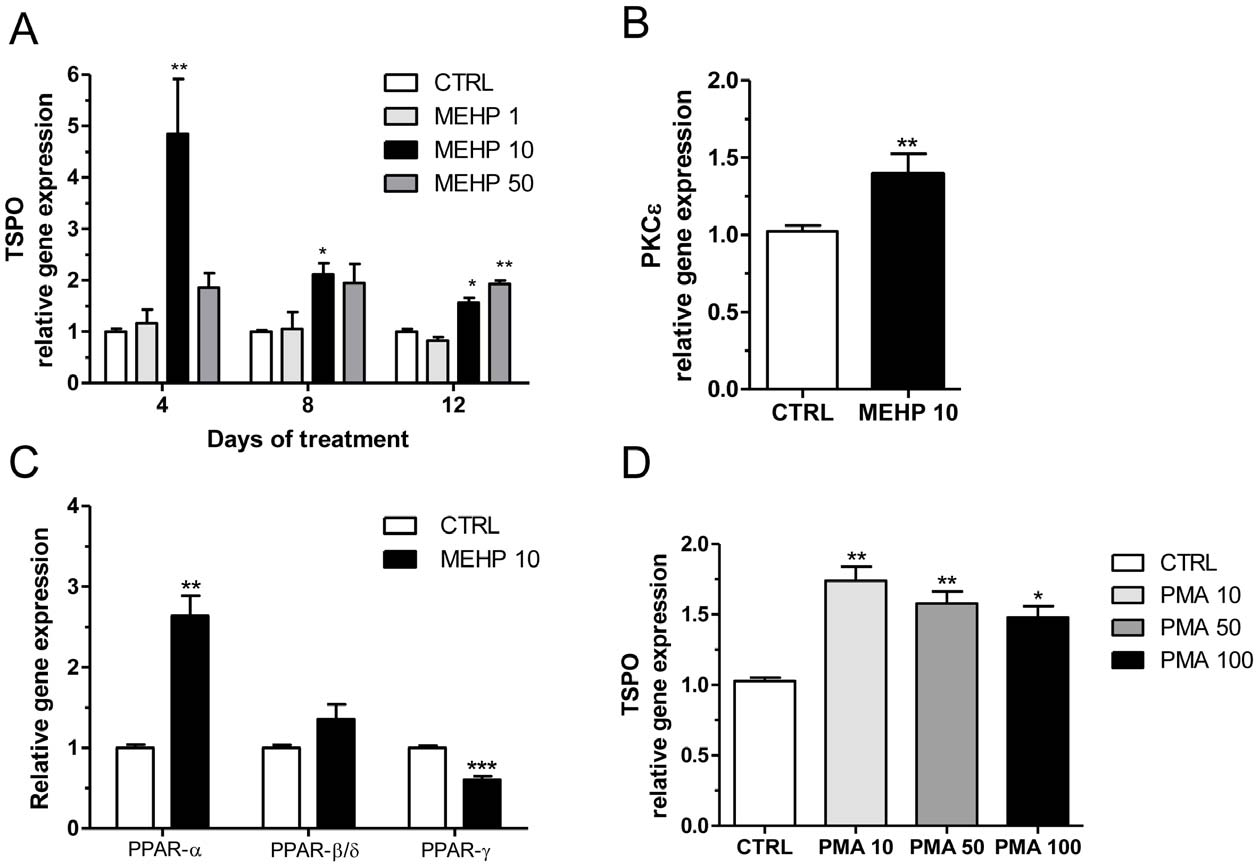
Effect of MEHP and PMA on the gene transcription of TSPO, PKCε, PPAR-α, PPAr-β/δ, and PPAR-γ. (A) Dose response of TSPO gene expression after 4, 8 and 12 days of treatment with 1, 10, and 50 µM MEHP. (B) Effect of 10 µM MEHP on PKCε gene expression after 4 days of treatment. (C) Effect of 10 µM MEHP on PPAR-α, PPAR-β/δ, and PPAR-γ gene expression after 4 days of treatment. (D) Effect of 10, 50, or 100 10 nM PMA on TSPO gene expression after 4 days of treatment. Cells were seeded for 24 h before treatment with MEHP (A–C) or PMA (D) and collected after the indicated time points (A) or after 4 days (B–D). qRT-PCR results are normalized to RPS18, expressed in terms of mean and S.E.M. calculated from 3 independent experiments, and presented as fold increase or decrease compared to control. One-way ANOVA followed by Bonferroni's post hoc test (A) or Student's *t*-test (B–D) was used to calculate statistical significance; *p<0.05, **p<0.01, ***p<0.001.

Based on our finding of increased *TSPO* transcription during human adipocyte differentiation, we investigated the effect of MEHP on *TSPO* gene transcription. While treatment with 1 µM MEHP produced no effect (Fig. 2B, n = 3), treatment with 10 µM MEHP for 4, 8, and 12 days resulted in approximately 4.5-fold (p<0.01; n = 3), 2.1-fold (p<0.05; n = 3), and 1.6-fold (p<0.05; n = 3) increases in TSPO mRNA levels, respectively (Fig. 2A). Treatment with 50 µM MEHP resulted in an approximately 2-fold increase in TSPO mRNA levels after 12 days of treatment (p<0.01; n = 3; Fig. 2B). Since treatment with 10 µM MEHP for 4 days resulted in the greatest increase in TSPO mRNA expression levels, this dose and time were chosen for subsequent experiments.

### MEHP induces the transcription of PKCε, PPAR-α, and β/δ, and reduces PPAR-γ

We have previously reported that PKCε controls *TSPO* expression through a MAPK pathway (Raf-1/ERK2) in steroidogenic cells [27]. We now examined whether MEHP was able to modify PKCε mRNA levels, as well as PPAR-α, PPAR-β/δ, and PPAR-γ in preadipocytes. Treatment with 10 µM MEHP for 4 days induced approximately 1.4-fold and 2.7-fold increases in PKCε (p<0.01; n = 3; Fig. 2B) and PPAR-α (p<0.01; n = 3; Fig. 2C) mRNA levels, respectively. On the other hand, PPAR γ mRNA levels decreased 36% (p<0.001; n = 3; Fig. 2C), and PPAR β/δ transcription was not significantly modified (Fig. 2C) by MEHP, compared to control (n = 3).

### PMA increases TSPO mRNA levels

We tested the effect of the PKCε agonist PMA on TSPO mRNA levels in SW 872 cells. Treatment with 10, 50, and 100 nM PMA for 4 days increased TSPO mRNA levels by approximately 1.8-fold, 1.6-fold, and 1.5-fold, respectively, compared to control (p<0.01, p<0.01 and p<0.05; n = 3; Fig. 2D).

### MEHP induces the transcription of differentiation markers

**Figure 3:**
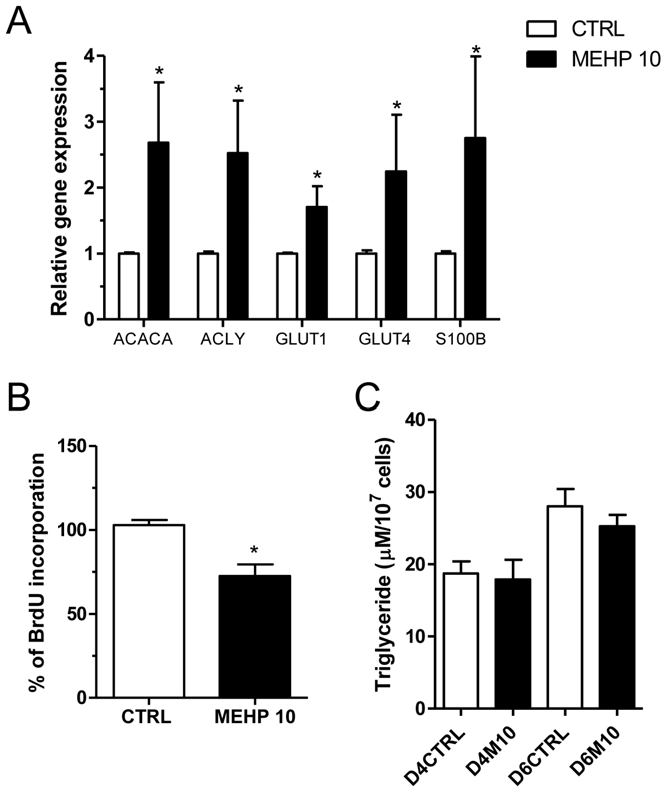
MEHP induces the differentiation of SW 872 cells. (A) qRT-PCR products of ACACA, ACLY, GLUT1, GLUT4 and S100B mRNA normalized to RPS18 and presented as fold increase or decrease compared to control, 4 days after treatment with 10 µM MEHP. (B) Proliferation assay. Cells were seeded for 24 h before treatment with 10 µM MEHP and the assay was performed as described in section 2.4 (C) Cellular triglyceride content assay. Cells were seeded for 24 h before treatment with 10 µM MEHP and collected after 4 or 6 days to assay triglyceride content. D0, D4CTRL, and D4M10 represent untreated cells at day 0, untreated cells at day 4, and MEHP-treated cells at day 4, respectively. Results are expressed in terms of mean and S.E.M., calculated from three independent experiments. Student's *t*-test was used to calculate statistical significance compared to control; *p<0.05.

To examine the effect of MEHP on SW 872 differentiation, we determined the mRNA levels of the following well-known differentiation markers: acetyl-CoA carboxylase alpha (ACACA) and adenosine triphosphate citrate lyase (ACLY), which are involved in the lipogenesis pathway, GLUT1 and GLUT4, which are involved in glucose transport, and S100B, a free fatty acid carrier that is highly expressed in the early stage of differentiation [34], [35], [36]. The effect of MEHP on C/EBP-α levels was not assessed, as C/EBP-α is expressed at high levels in terminally differentiated cells [37], while the effect of MEHP on TSPO is predominantly in the early phase of SW 872 cell differentiation. Treatment with 10 µM MEHP for 4 days caused increases in the mRNA levels of these differentiation markers, compared to control, as follows: ACACA, approximately 2.8-fold; ACLY, approximately 2.6-fold; GLUT1, approximately 1.7-fold; GLUT4, 2.4-fold; S100B, approximately 1.8-fold (p<0.05; n = 3; Fig. 3A).

### MEHP exposure inhibits BrdU incorporation

Since proliferation and differentiation have an inverse relationship during adipocyte differentiation [38], we assessed the effect of MEHP on SW 872 cell proliferation using the BrdU assay. We observed a 27.3% reduction (p<0.05) of BrdU incorporation into SW 872 preadipocytes upon MEHP treatment, compared to control cells (Fig. 3B), indicating that the compound has an inhibitory effect on cell proliferation.

### MEHP has no effect on triglyceride content

Preadipocytes have a fibroblast-like morphology, but become spherical and filled with lipid droplets upon differentiation into adipocytes. Four days after seeding, SW 872 cells still exhibit the morphological characteristics of preadipocytes, and are poor in lipid content [39], [40]. We quantified the triglyceride content of SW 872 cells after treatment with MEHP for 4 days, and at 6 days to exclude a possible delayed effect on the triglyceride production machinery following MEHP administration. The results obtained showed no change in cellular triglyceride content after treatment with MEHP, compared to control cells (Fig. 3C).

### MEHP regulates TSPO mRNA levels acting through PPAR-α and PPAR-β/δ

We have previously shown that the effect of MEHP on TSPO mRNA levels was mediated through the action of PPAR-α in MA-10 Leydig cells [24]. We therefore investigated the role of PPARs in mediating the effect of MEHP on TSPO transcription in SW 872 cells. PPAR-α, β/δ and γ were silenced using specific siRNAs, and the levels of TSPO mRNA were determined following treatment with 10 µM MEHP for 4 days.

**Figure 4:**
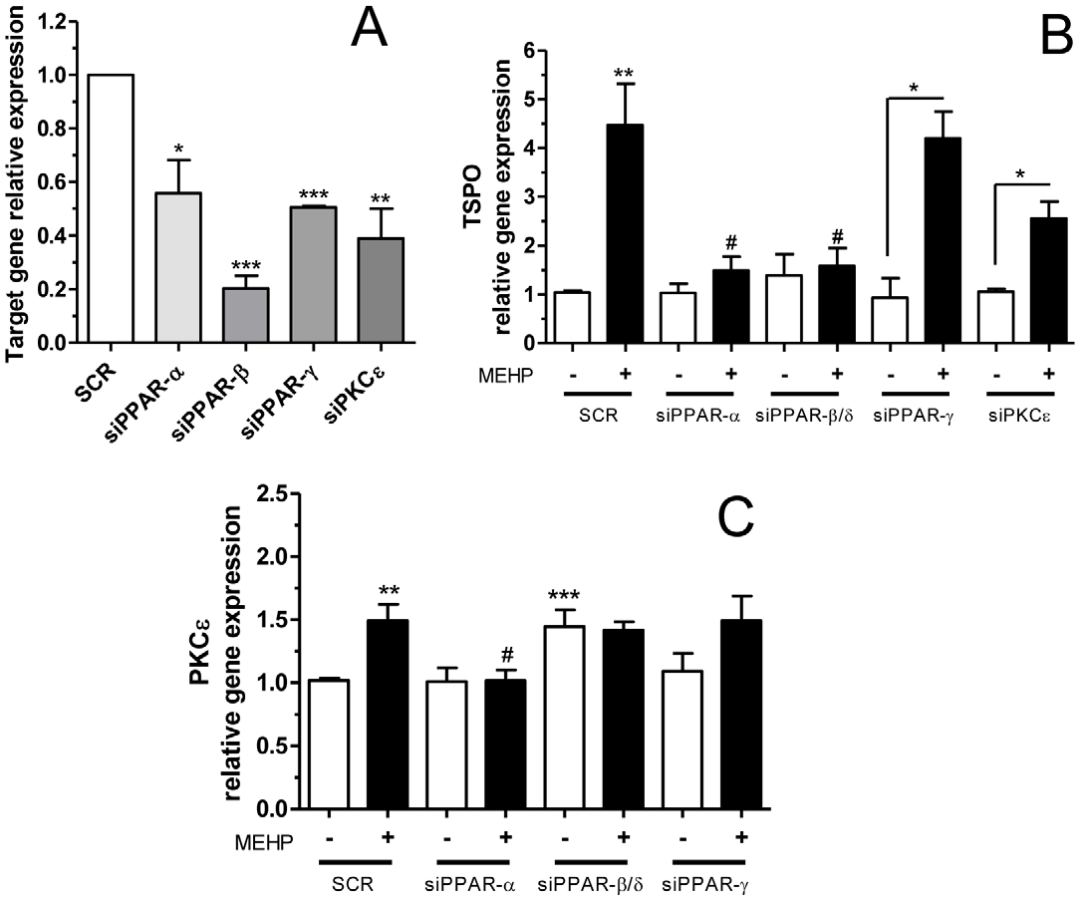
The effect of MEHP on TSPO gene expression is mediated by PPAR-α and PPAR-β/δ , and on PKCε gene expression by PPAR-α. (A) PPAR-α, PPAR-β/δ, PPAR-γ and PKC mRNA levels are greatly reduced following treatment with gene-specific siRNAs compared to treatment with scrambled siRNA. (B) Effect of gene-specific siRNA treatment on TSPO transcription, with or without (+/−) MEHP, compared to similar treatment with scrambled siRNA (SCR). (C) Effect of gene-specific siRNA treatment on PKCε transcription, with or without (+/−) MEHP compared to similar treatment with scrambled siRNA (SCR). Cells were seeded for 24 h before treatment with MEHP, and then transfected with siRNA specific to PPAR-α, PPAR-β/δ, PPAR-γ, and PKCε knockdown. Cell lysates were collected after 4 days. qRT-PCR results are expressed in terms of mean and S.E.M., calculated from three independent experiments. Student's *t*-test was used to calculate statistical significance compared to scrambled siRNA control in the absence (*) or presence (#) of MEHP; *p<0.05, **p<0.01, ***p<0.001, ^#^p<0.05.

We initially verified the knockdown of PPAR gene expression by the various siRNAs, and observed, on average, a 40–80% reduction in PPAR mRNA levels, as shown in Fig. 4A. TSPO mRNA levels were reduced by 75% in samples transfected with PPAR-α siRNA and treated with MEHP, compared to cells transfected with scrambled siRNA and treated with MEHP (p<0.05; Fig. 4B). We observed a similar reduction in TSPO mRNA levels in cells transfected with PPAR-β/δ siRNA and treated with MEHP (p<0.05; Fig. 4B), while no change was observed in TSPO mRNA levels in cells transfected with PPAR-γ or PKCε siRNAs and treated with MEHP (Fig. 4B). These observations suggest that the effect of MEHP on TSPO gene transcription is mediated by PPAR-α and β/δ.

### MEHP acts on PKCε gene transcription through PPAR-α

We demonstrated earlier that PKCε mRNA levels increased upon treatment with MEHP (Fig. 2B). We examined whether the PPARs were mediating this effect. Among the PPARs tested, silencing of PPAR-α alone resulted in a reduction of PKCε mRNA by 50% (p<0.05) after treatment with MEHP, compared to control cells transfected with scrambled siRNA and treated with MEHP (Fig. 4C).

### MEHP reduces TSPO protein expression

**Figure 5:**
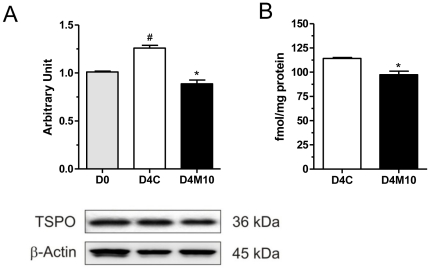
MEHP treatment results in decreased TSPO protein level. (A) Densitometric analysis of TSPO immunoblot. Cells were seeded for 24 h before treatment with 10 µM MEHP and collected at day 0 and after 4 days. D0, D4C, and D4M10 represent untreated cells at day 0, untreated cells at day 4, and MEHP-treated cells at day 4, respectively. (B) Saturation binding assay. Cells were seeded for 24 h before treatment with MEHP and collected after 4 days. D4C, and D4M10 represent untreated cells and MEHP-treated cells at day 4, respectively. Results are expressed in terms of mean and S.E.M., calculated from two independent experiments. Student's *t*-test was used to calculate statistical significance compared to control (*p<0.05), or to day 0 (^#^p<0.05).

Changes in TSPO protein levels were examined by immunoblot analyses and the [^3^H]PK 11195 ligand binding assay. Immunoblot analysis of SW 872 cell lysates at D0 showed the presence of the TSPO dimer at 36 kDa [41], [42]. A significant increase in TSPO levels (p<0.05) was observed in untreated cells after 4 days of differentiation (Fig. 5A), and this differentiation-induced increase in TSPO protein levels was inhibited by treatment with MEHP treatment (Fig. 5A). Radioligand binding studies followed by Scatchard analysis of the saturation isotherms confirmed these results, showing a significant reduction in the maximal number of binding sites (B_max_) in MEHP treated cells (93.9±1.4 fmol/mg protein), compared to control cells (114.2±1.0 fmol/mg protein; p<0.05; Fig. 5B); however, no significant changes in the affinity (K_d_) of TSPO for the ligand were observed.

### TSPO knockdown increases the levels differentiation markers

**Figure 6:**
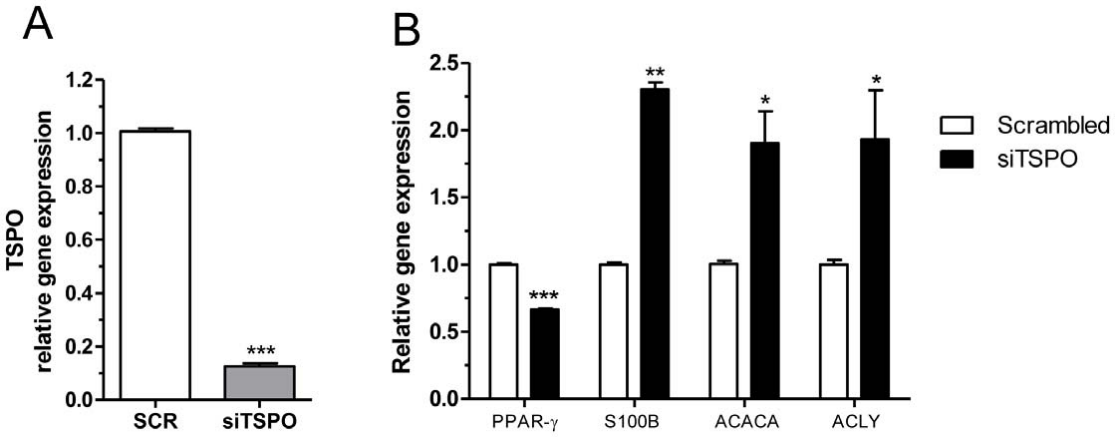
TSPO gene knockdown decreases PPAR-γ transcription and increases transcription of S100B, ACACA, and ACLY. (A) TSPO mRNA levels following treatment with gene-specific siRNA, compared to similar treatment with scrambled siRNA. (B) Effect of gene-specific siRNA treatment on PPAR-γ, S100B, ACACA, and ACLY mRNA levels compared to treatment with scrambled siRNA. Cells were transfected with siRNA as described in section 2.5, and collected after 4 days. qRT-PCR results are expressed in terms of mean and S.E.M., calculated from three independent experiments. Student's *t*-test was used to calculate statistical significance compared to scrambled siRNA treatment; * p<0.05, **p<0.01, ***p<0.001.

Based on our observation of TSPO downregulation upon MEHP treatment during differentiation, we examined the effect of TSPO knockdown on mRNA levels of PPAR-γ and other differentiation markers. Silencing of TSPO expression using TSPO siRNA was highly effective (∼87%; Fig. 6A). After 4 days in culture, silencing of TSPO resulted in 34% reduction in PPAR-γ mRNA levels (p<0.001), while the mRNA levels of S100B, ACACA, and ACLY showed increases of approximately 2.3-fold (p<0.01), approximately 1.9-fold (p<0.05), and approximately 1.9-fold (p<0.05), respectively, compared to control (Fig. 6B).

## Discussion

The impact of plasticizers on human health is one of the main issues in the modern era, due to their extensive use in the industrial production. Although initially regarded as exhibiting low toxicity [43], [44], phthalates were later shown to be carcinogenic [45], [46] and teratogenic [47], to affect fertility and litter size [47]–[49], and to exert adverse effects on the reproductive system [1]. Indeed, we previously reported that exposure to DEHP exerts suppressive effects on testosterone production in the rat [50], and that MEHP acts as a mitochondrial toxicant and lipid metabolism disruptor in Leydig cells [51]. The purpose of the present work was to evaluate the effect of MEHP on the differentiation of human SW 872 preadipocytes, and to determine the involvement of TSPO and the PPAR signaling pathway in this process.

It was previously demonstrated that MEHP directly activates PPAR-γ and promotes adipogenesis in murine 3T3-L1 cells [52]. After 4 days of treatment with 10 µM MEHP, we observed an increase in the mRNA levels of the early differentiation markers ACACA and ACLY, key enzymes for de novo lipogenesis, as well as in the mRNA levels of GLUT1, GLUT4, and S100B. This effect on gene expression coincided with a reduction in cellular proliferation; however, the cellular triglyceride content was not modified. The absence of any change in the triglyceride content is likely due to the well-documented poor lipid content of this cell line [39], especially in the early phase of differentiation [40]. Nevertheless, these data taken together indicate that MEHP exerts a differentiating effect in human preadipocytes in agreement with the results obtained with 3T3-L1 mouse preadipocytes [52].

Treatment with 10 µM MEHP for 4 days significantly increased the transcription of *TSPO* mRNA, with the maximal increase observed at 4 days, and a progressive decline thereafter. This suggests that the effect of MEHP is transient, with a maximal effect at or around day 4. We have previously demonstrated that PKCε affects TSPO expression through the MAPK pathway (Raf-1/ERK2) in steroidogenic cells [27]. In the current study, we found that TSPO mRNA increased in SW 872 cells after 4 days of treatment with PMA, a PKCε agonist. However, gene silencing of PKCε followed by treatment with MEHP did not produce a significant decrease in TSPO mRNA expression, indicating that the transcriptional effect of MEHP is PKCε–independent in SW 872 cells. Moreover, data obtained by using a PKCε translocation inhibitor peptide at different concentrations, also confirmed that PKCε has no role in mediating the MEHP-induced TSPO mRNA increased expression in SW 872 cells (data not shown). On the contrary, further gene silencing studies demonstrated that the transcriptional effect of MEHP on TSPO is mediated by PPAR-α and PPAR-β/δ. The relationship between the PKC family and the PPARs is not yet clear, although peroxisome proliferators have been shown to stimulate the activity of protein kinase C in vitro [53]. In this study, we have demonstrated that the PPAR agonist MEHP directly affects the transcription of PKCε mRNA, and that knocking down PPAR-α can block this effect. It is known that PPAR ligands regulate the transcription of their own receptors; for example, PPAR-α agonists have been shown to upregulate PPAR-α mRNA [54], [55], while PPAR-γ agonists downregulate PPAR-γ mRNA [56], [57]. We observed an increase in PPAR-α mRNA and a decrease in PPAR-γ mRNA following MEHP treatment. In light of the decrease in PPAR-γ mRNA levels observed in SW 872 cells during differentiation [32], our observed reduction in PPAR-γ mRNA is further proof of MEHP-induced differentiation.

Using an immunoblot assay, we observed a decrease in TSPO protein expression following MEHP treatment. To confirm this observation, we also carried out a saturation binding assay for TSPO using the radiolabeled ligand PK 11195. Scatchard analysis of the saturation isotherms confirmed a significant decrease in the number of PK 11195 ligand binding sites in MEHP-treated cells, compared to the number in control cells. Although the changes observed in TSPO levels range from 20–35% one should consider that TSPO is a mitochondrial protein thought by many as a housekeeping gene. In mitochondria, TSPO comprises 2% of the OMM protein and is part of the mitochondrial transition pore [25], [26]. As noted earlier TSPO has been implicated in mitochondrial respiration, lipid import, protein import, biogenesis and apoptosis [25], [26]. Moreover, mitochondrial function has been closely linked to adipocyte differentiation and homeostasis [58]. Thus, even small changes in TSPO expression could have a major impact on adipogenesis.

To follow up on these observations, we analyzed the effect of siRNA silencing of the *TSPO* gene on the mRNA levels of the differentiation markers. Interestingly, the silencing of TSPO results in an increase the levels of differentiation markers of the enzymes and proteins directly involved in the formation of the lipid mass for storage. It has been previously shown that TSPO mRNA expression increases during the differentiation of mouse preadipocytes, and decreases when the process is completed [29]. In this study, we observe that MEHP treatment results in an increase in TSPO mRNA levels; however, this increase is not maintained during differentiation, and a progressive reduction of the protein levels is seen, as in mature adipose tissue.

**Figure 7:**
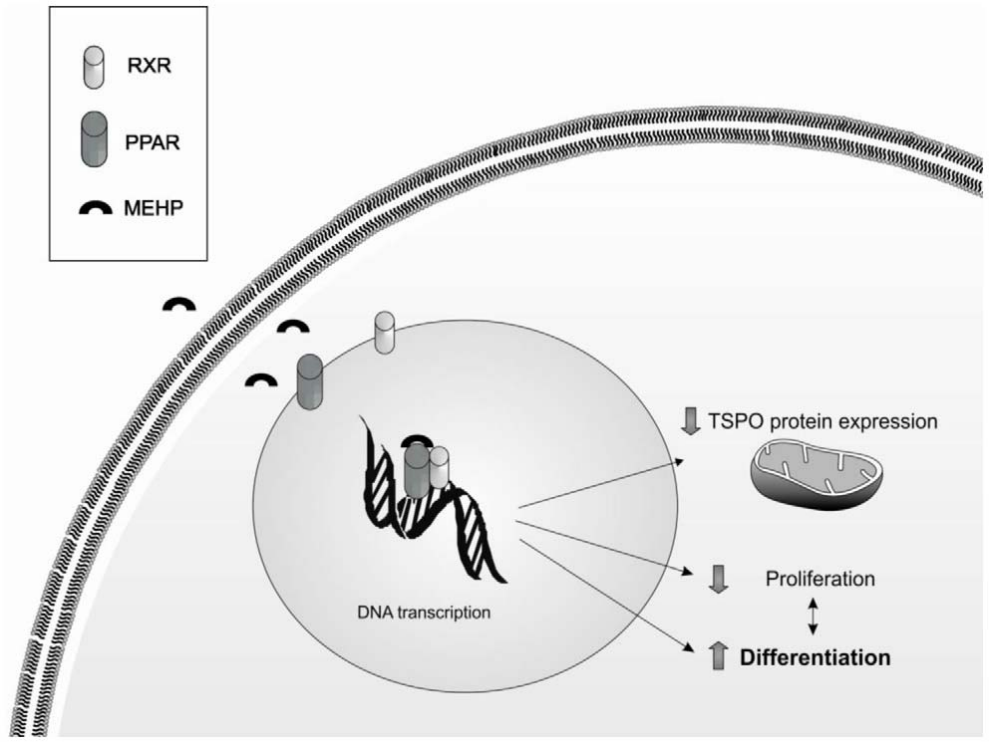
Effects of MEHP on SW 872 human preadipocytes. MEHP enhances differentiation, inhibits cellular proliferation, and decreases mitochondrial TSPO expression in SW 872 cells.

In conclusion, our observations taken together indicate that MEHP acts as a differentiating agent in human adipocytes, specifically in the early phase of the differentiation process, where SW 872 cells seem to be more sensitive to the endocrine disruptor (Fig. 7). Our data also suggest that TSPO could be considered an important player in the differentiation process, whose presence is essential for adipocyte development. To our knowledge, this is a newly identified function of TSPO in cell differentiation and in the maintenance of the balance between lipid trafficking and metabolism. Further studies are needed to fully characterize the relationship between PPAR-γ and TSPO, in the light of the observation that PPAR-γ mRNA transcription is affected by knocking down TSPO.
